# Corneal confocal microscopy differentiates patients with Parkinson’s disease with and without autonomic involvement

**DOI:** 10.1038/s41531-022-00387-8

**Published:** 2022-09-09

**Authors:** Ning-Ning Che, Shuai Chen, Qiu-Huan Jiang, Si-Yuan Chen, Zhen-Xiang Zhao, Xue Li, Rayaz A. Malik, Jian-Jun Ma, Hong-Qi Yang

**Affiliations:** 1 Department of Neurology, Henan Provincial People’s Hospital, School of Clinical Medicine, Henan University, 450003 Zhengzhou, China; 2 Department of Neurology, Affiliated BenQ Hospital of Nanjing Medical University, 210019 Nanjing, China; 3 Department of Medicine, Weill Cornell Medicine-Qatar, Doha, Qatar; 4 Division of Cardiovascular Sciences, School of Medical Sciences, University of Manchester, Manchester, UK; 5 Department of Neurology, People’s Hospital of Zhengzhou University, School of Clinical Medicine, Zhengzhou University, 450003 Zhengzhou, China

**Keywords:** Diagnostic markers, Neurological disorders

## Abstract

Autonomic dysregulation in Parkinson’s disease (PD) can precede motor deficits and is associated with reduced quality of life, disease progression, and increased mortality. Objective markers of autonomic involvement in PD are limited. Corneal confocal microscopy (CCM) is a rapid ophthalmic technique that can quantify small nerve damage in a range of peripheral and autonomic neuropathies. Here we investigated whether CCM can be used to assess autonomic symptoms in PD. Based on the scale for outcomes in Parkinson’s disease for autonomic symptoms (SCOPA-AUT), patients with PD were classified into those without autonomic symptoms (AutD-N), with single (AutD-S), and multiple (AutD-M) domain autonomic dysfunction. Corneal nerve fiber pathology was quantified using CCM, and the relationship with autonomic symptoms was explored. The study enrolled 71 PD patients and 30 control subjects. Corneal nerve fiber density (CNFD), corneal nerve branch density (CNBD), corneal nerve fiber length (CNFL), and CNBD/CNFD ratio were lower in PD patients with autonomic symptoms compared to those without autonomic symptoms. Autonomic symptoms correlated positively with CNFD (*r* = −0.350, *p* = 0.004), and were not related to Levodopa equivalent daily dose (*r* = 0.042, *p* = 0.733) after adjusting for age, disease severity, disease duration or cognitive function. CCM parameters had high sensitivity and specificity in distinguishing patients with PD with and without autonomic symptoms. PD patients with autonomic symptoms have corneal nerve loss, and CCM could serve as an objective ophthalmic imaging technique to identify patients with PD and autonomic symptoms.

## Introduction

Parkinson’s disease (PD) is a complex neurological disorder that can present with motor and non-motor symptoms^1^, although motor symptoms such as tremors and bradykinesia are the main reason for patients to seek medical advice^2^. Rapid eye movement sleep behavior disorder, olfactory deficits, and signs and symptoms of autonomic dysregulation can precede motor deficits and could therefore be targeted as prodromal or diagnostic biomarkers in PD^3,4^.

Although PD is traditionally regarded as a central neurodegenerative disease (CNS), peripheral nerve involvement is increasingly recognized^5,6^. Large fiber (Aα/β fibers) involvement may be related to levodopa administration, but small fiber (Aδ and C fibers) neuropathy is thought to be intrinsic to the neurodegenerative process in PD^7^. Moreover, epidemiological and experimental studies suggest that the dysfunctional autonomic innervation in the gut, heart, and skin may provide a route by which Parkinson’s disease pathology spreads both to and from the CNS^8^. Large fiber neuropathy is usually diagnosed with nerve conduction studies, and small fiber neuropathy can be assessed in skin biopsy^9^. Reliable and easy-to-use tests of autonomic integrity may be critical in early diagnosis and to assess the impact of interventions that prevent neurodegeneration in PD.

Multiple studies have shown that corneal confocal microscopy (CCM) can be used to quantify corneal nerve loss, has good diagnostic utility for diabetic neuropathy^10^, and predicts the development of diabetic neuropathy^11^. Furthermore, corneal nerve loss has very high sensitivity and specificity and has been related to the severity of diabetic autonomic neuropathy^12^. An increasing number of studies have shown evidence of corneal nerve loss in PD patients^13^, which has been associated with motor and non-motor symptoms^14,15^. Furthermore, in a longitudinal study, Lim et al showed that greater corneal nerve loss was associated with more rapid motor progression in a cohort of patients with PD^14^. We have also recently shown that the severity of corneal nerve loss was associated with the severity of cognitive dysfunction in PD^15^.

In the present study, the relationship between corneal nerve loss, quantified using CCM, and the severity of autonomic symptoms was evaluated with the scale for outcomes in Parkinson’s disease for autonomic symptoms (SCOPA-AUT). The diagnostic utility of CCM for autonomic symptoms in PD was also established.

## Results

### Clinical profiles

Of 84 PD patients enrolled, 71 underwent complete investigations. Eight patients with impaired glucose tolerance, 3 with corneal disease, and 2 with a suspected diagnosis of multiple system atrophy were excluded. The age of the PD group (44.62% male) was 62.59 ± 7.75 years old, with an age at onset 58.69 ± 8.12 years and a disease duration of 3 (2, 4) years. The average H-Y stage of the PD group was 2 (1,3) and was comprised of H-Y I (21, 29.58%), H-Y II (21, 29.58%), H-Y III (14, 19.72%), and H-Y IV (15, 21.13%), respectively. In the control group (*n* = 30), 53.33% were male, with an average age of 62.43 ± 6.16 years.

### Autonomic symptoms

**Fig. 1:**
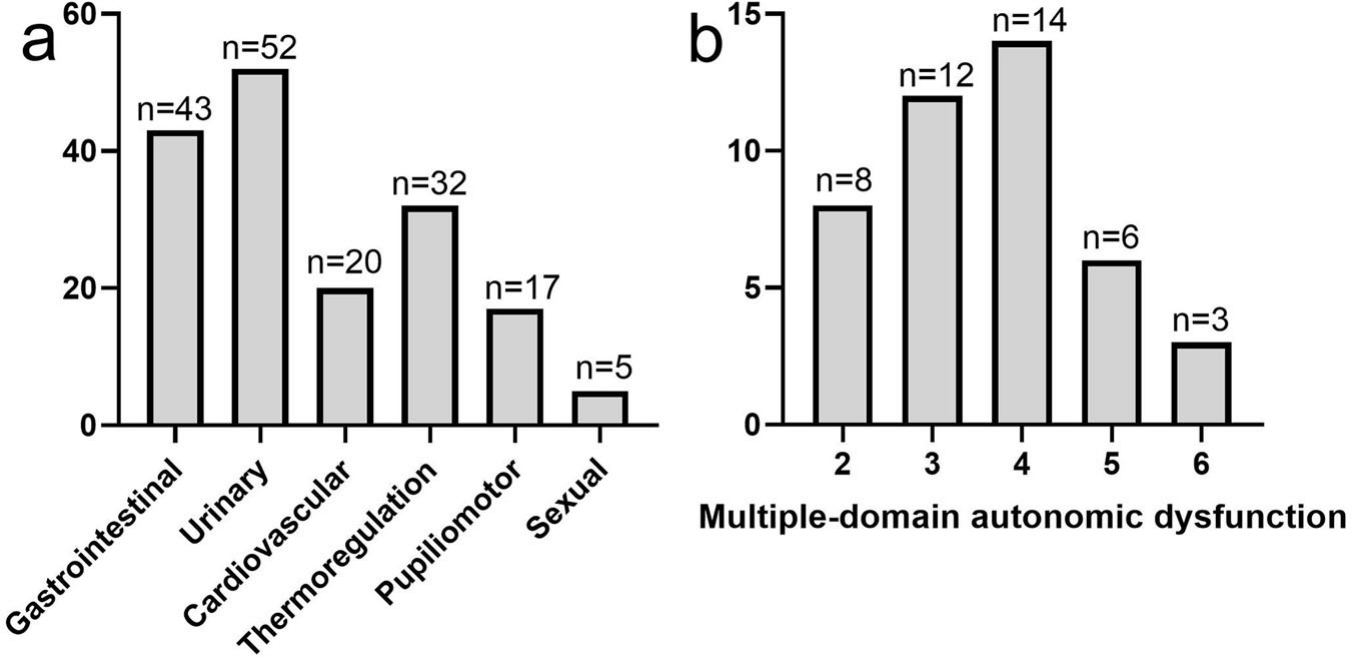
Different autonomic symptoms in PD patients. The frequency of different autonomic symptoms in PD patients (**a**), and the frequency of domains involved in the AutD-M group (**b**).

Of the 71 PD patients, 14 (19.7%) had no autonomic symptoms (AutD-N), 14 (19.7%) had autonomic symptoms in one domain (AutD-S), and 43 (60.6%) had autonomic symptoms in two or/more domains (AutD-M). The demographic and clinical profiles of each subgroup are presented in Table 1. The SCOPA-AUT score in each group was 0 (AutD-N), 6.93 ± 2.87 (AutD-S), and 15.93 ± 6.47 (AutD-M), respectively. The relative frequency of domains affected: urinary (52), gastrointestinal (43), thermoregulatory (32), cardiovascular (20), pupillomotor (17), and sexual (5) is depicted in Fig. 1a. Urinary complaints were primarily incontinence, incomplete emptying and increased frequency, whilst gastrointestinal complaints included constipation, swallowing difficulties and sialorrhea. Other autonomic symptoms include hyperhidrosis (thermoregulatory), light-headiness when standing up (cardiovascular), and oversensitivity to bright light (pupillomotor). Erectile dysfunction and retrograde ejaculation in men and reduced vaginal lubrication and orgasm were reported in females. Orthostatic hypotension was reported in 22 patients (30.98%): 4 (28.57%) in AutD-S group and 18 (41.86%) in the AutD-M group. In the AutD-M group, the numbers of patients with involvement of 2, 3, 4, 5, and 6 domains were 8, 12, 14, 6, and 3, respectively (Fig. 1b).

**Table 1:** Demographic characteristics of participants.

	Controls (*n* = 30)	No autonomic dysfunction (*n* = 14)	Single-domain autonomic dysfunction (*n* = 14)	Multiple-domain autonomic dysfunction (*n* = 43)
Age, years	62.43 ± 6.16	60.21 + 5.70	62.71 ± 7.71	63.33 ± 8.32
Sex M/F	16/14	8/6	5/9	18/25
Disease duration, years	NA	2.0 (1.0, 3.0)	2.18 ± 1.45^a^*	3.0 (2.0, 5.0)^b^**
H-Y Stage	NA	1.0 (1.0, 2.0)	2.0 (1.0, 2.25)^a^*	3.0 (2.0, 4.0)^b^***
LEDD, mg	NA	422.50 ± 152.56	458.04 ± 112.29	500 (412.500, 750.00)
UPDRS-I	NA	7.50 ± 3.67	9.0 (6.75, 12.0)	14.33 ± 7.15^b^**
UPDRS-II	NA	9.79 ± 3.91	10.50 ± 5.13^a^**	17.47 ± 7.33^b^**
UPDRS-III	NA	19.71 ± 10.20	25.86 ± 13.15	37.40 ± 18.72^b^**
UPDRS-total	NA	37.00 ± 12.57	46.57 ± 16.19^a^*	69.19 ± 20.50^b^***
HAMA	NA	14.93 ± 6.58	16.43 ± 5.30	17.26 ± 6.15
HADA	NA	12.21 ± 6.57	12.07 ± 6.62	12.63 ± 5.29
MoCA	NA	25.57 ± 2.85	23 (12.25, 26.00)	20.40 ± 6.25^b^*
CNFD no./mm^2^	34.33 ± 3.78	30.88 ± 2.42	27.87 ± 3.11	23.63 ± 3.93
CNBD no./mm^2^	24.58 ± 8.23	40.22 (34.95, 58.05)	37.52 ± 7.61	25.92 ± 10.60
CNFL mm/mm^2^	15.86 ± 2.27	17.54 ± 2.03	15.74 ± 1.56	12.85 ± 2.55
CNBD/CNFD	0.72 ± 0.24	1.47 ± 0.45	1.36 ± 0.32	1.03 (0.69, 1.55)

Numbers are expressed as mean ± SD or median (interquartile range). For variables following a normal distribution, analysis of variance (ANOVA) with Bonferroni as post hoc test was used for multi-group comparison. For variables following a non-normal distribution or non-homoscedasticity, nonparametric Kruskal–Wallis test was used for multi-group comparison. Categorical variables were compared with Chi-square tests and Fisher’s exact tests.

*NA* not available, *UPDRS* unified Parkinson’s disease rating scale, *LEDD* levodopa equivalent daily dose, *SCOPA-AUT* the scale for outcomes in PD for autonomic symptoms, *HAMA-14* the 14-item Hamilton anxiety rating scale, *HADA-24*, the 24-item Hamilton depression rating scale, *MoCA* Montreal cognitive assessment, *CNFD* corneal nerve fiber density, *CNBD* corneal nerve branch density, *CNFL* corneal nerve fiber length.

*P < 0.05, **P < 0.01, ***P < 0.001.

^a^Differences between AutD-S and AutD-M.

^b^Differences between AutD-N and AutD-M.

### Corneal nerve fiber parameters

**Fig. 2:**
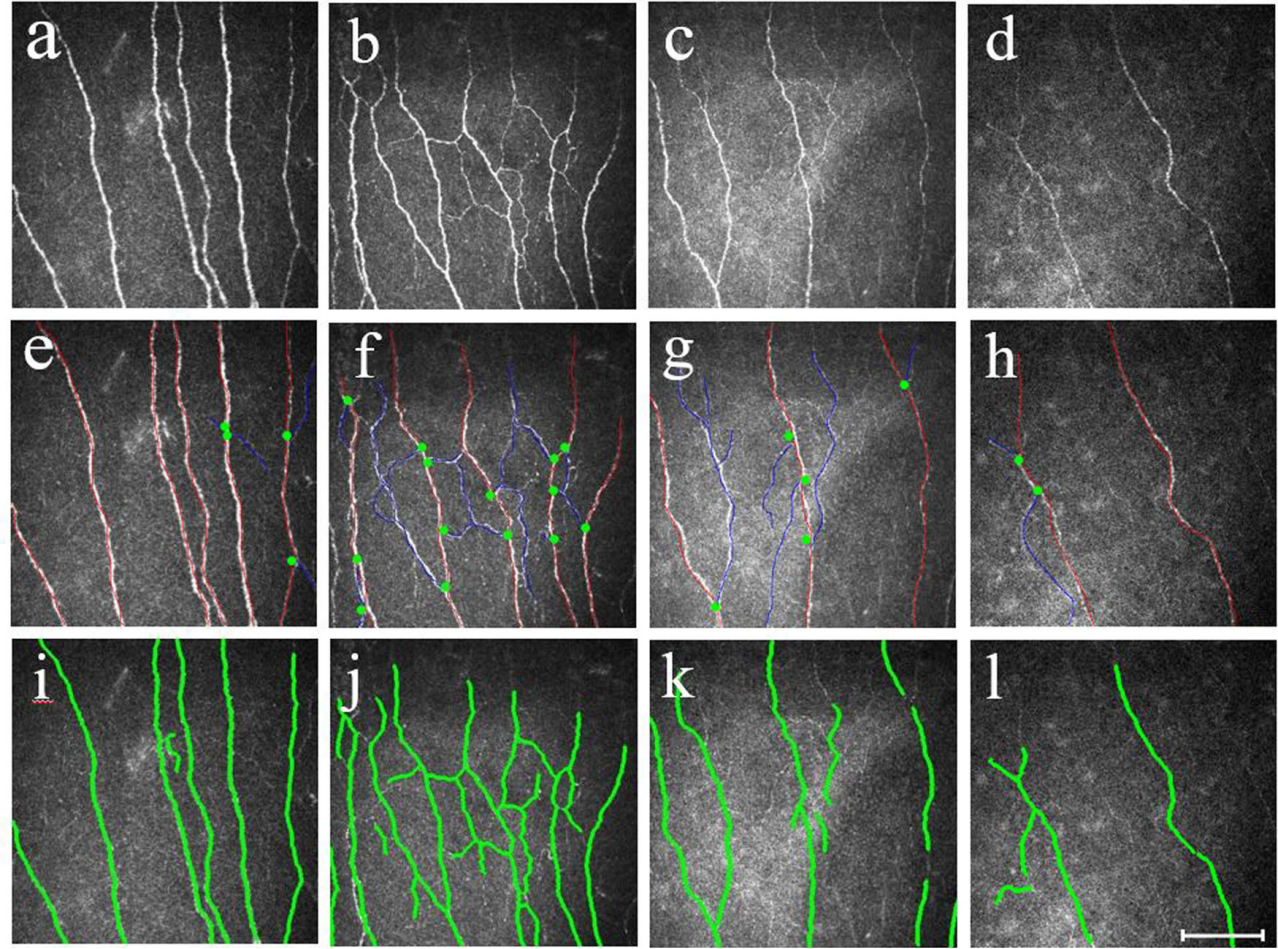
Representative CCM images in healthy controls, AutD-N, AutD-S, and AutD-M. Corneal nerve fibers are beaded, linear homogeneous and highly reflective (**a**–**d**). Nerve fiber trunks are highlighted in red, green dots indicate the origin of the branches, and blue and red lines combined indicate the corneal nerve fiber length (**e**–**h**). Pictures (**e**–**h**) were analyzed with the manual software (CCMetrics) and images (**i**–**l**) were marked with the automated version (ACCMetrics). CCM corneal confocal microscopy, PD Parkinson’s disease, AutD-N patients with no autonomic symptoms, AutD-S patients with single-domain autonomic symptoms, AutD-M patients with multiple-domain autonomic symptoms. Scale bar = 100 um.

**Fig. 3:**
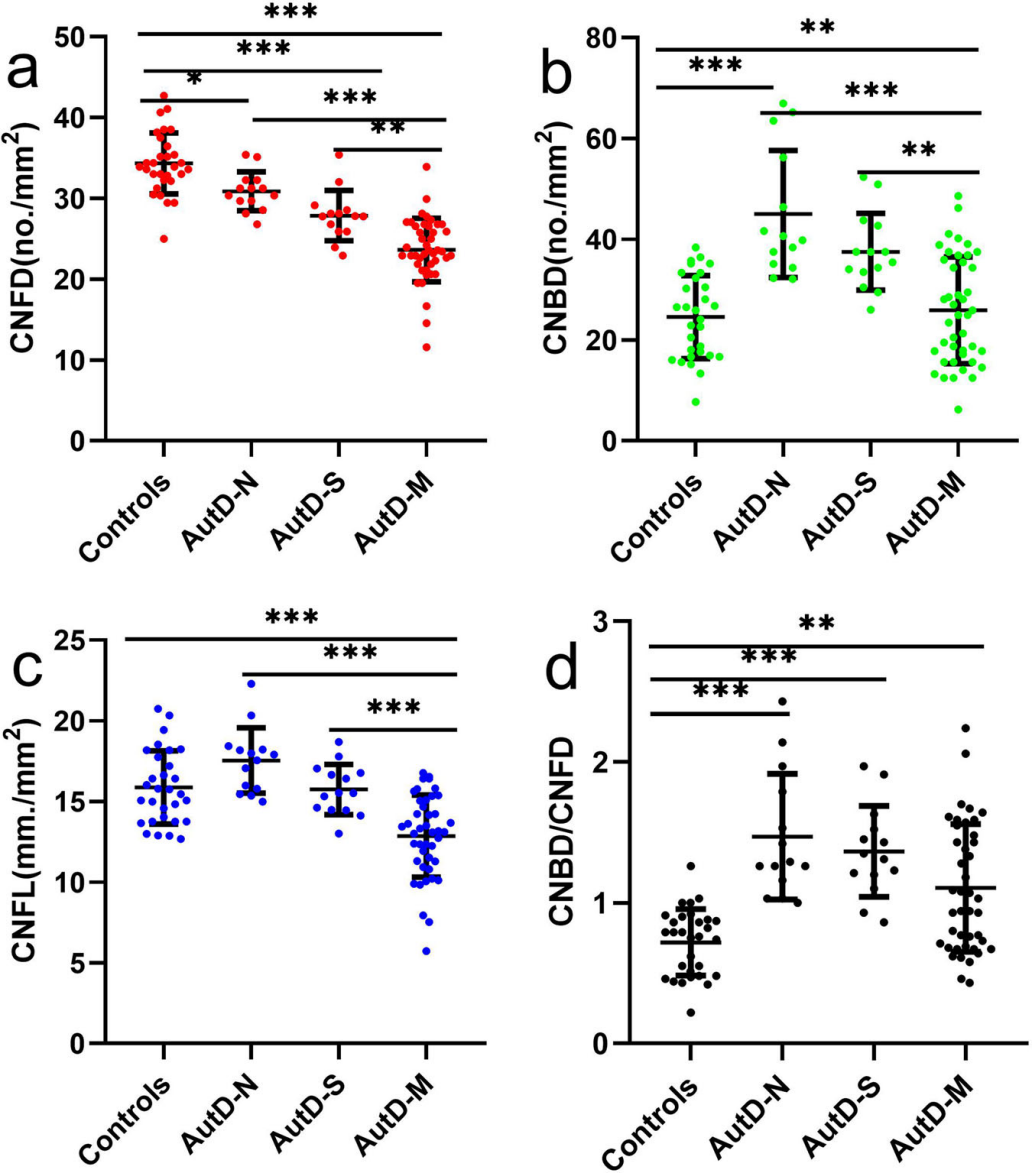
CCM measurements in controls and PD patients with varying degree of autonomic dysfunction. CNFD, CNBD, CNFL, and CNBD/CNFD ratio in AutD-N, AutD-S, and AutD-M groups were compared with controls (**a**–**d**). Errors bars represent mean ± standard deviation (*P < 0.05, **P < 0.01, ***P < 0.001). PD Parkinson’s disease, AutD-N patients with no autonomic symptoms, AutD-S patients with single-domain autonomic symptoms, AutD-M patients with multiple-domain autonomic symptoms, CNFD corneal nerve fiber density, CNBD corneal nerve branch density, CNFL corneal nerve fiber length.

There was a progressive loss of CNFD, CNBD, and CNFL with increasing severity of autonomic dysfunction (Figs. 2 and 3). CNFD in AutD-N (no./mm^2^, 30.88 ± 2.42 vs 34.33 ± 3.78; mean difference, 3.45; 95% CI, 0.29–6.59; *P* = 0.024, by one-way ANOVA), AutD-S (no./mm^2^, 27.87 ± 3.11 vs 34.33 ± 3.78; mean difference, 6.46; 95% CI, 3.31–9.61; *P* < 0.001, by one-way ANOVA) and AutD-M (no./mm^2^, 23.63 ± 3.93 vs 34.33 ± 3.78; mean difference, 10.70; 95% CI, 8.39–13.01; *P* < 0.001, by one-way ANOVA) groups were significantly lower compared to controls. CNFD in AutD-M was significantly lower than in patients with AutD-S (no./mm^2^, 23.63 ± 3.93 vs 27.87 ± 3.11; mean difference, 4.24; 95% CI, 1.25–7.23; *P* = 0.001) and AutD-N (no./mm^2^, 23.63 ± 3.93 vs 30.88 ± 2.42; mean difference, 7.25; 95% CI, 4.26–10.25; *P* < 0.001).

CNBD in AutD-N (no./mm^2^, 40.22(34.92, 58.05) vs 24.58 ± 8.23; *P* < 0.001, by Kruskal–Wallis test) and AutD-S (no./mm^2^, 37.52 ± 7.61 vs 24.58 ± 8.23; *P* = 0.003, by Kruskal–Wallis test) was significantly higher compared to controls. AutD-S [no./mm^2^, 37.52 ± 7.61 vs 40.22 (34.95, 58.05); *P* = 0.007] and AutD-M [no./mm^2^, 25.92 ± 10.60 vs 40.22 (34.95, 58.05); *P* < 0.001] had a lower CNBD compared to the AutD-N group.

CNFL was significantly lower in the AutD-M compared to the AutD-S (mm/mm^2^, 12.85 ± 2.55 vs 15.74 ± 1.56; mean difference, 2.89; 95% CI, 0.99–4.79; *P* < 0.001), AutD-N (mm/mm^2^, 12.85 ± 2.55 vs 17.54 ± 2.03; mean difference, 4.69; 95% CI, 2.79–6.59; *P* < 0.001), and control (mm/mm^2^, 12.85 ± 2.55 vs 15.86 ± 2.27; mean difference, 3.02; 95% CI, 1.55–4.48; *P* < 0.001) group.

The CNBD/CNFD ratio was higher in all three PD groups compared to controls, with no significant difference between the subgroups with PD.

### Correlations between autonomic symptoms and clinical and CCM parameters

**Fig. 4:**
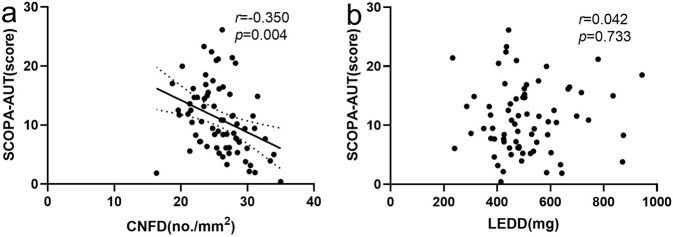
Association between SCOPA-AUT with CNFD and LEDD. **a**, **b** Partial correlation plots and standardized correlation cofficients between autonomic symptoms and CNFD. SCOPA-AUT the scale for outcomes Parkinson’s disease for autonomic symptoms, CNFD corneal nerve fiber density.

SCOPA-AUT was negatively associated with CNFD (*r* = −0.350, *P* = 0.004) after adjusting for confounders including age, disease severity, disease duration, and MoCA scores. There was no correlation between SCOPA-AUT and LEDD (*r* = 0.042, *p* = 0.733) (Fig. 4). CNFD (23.81 ± 2.95 vs 26.55 ± 4.83; *t* = 2.202, *P* = 0.031) was significantly lower, while CNBD (28.65 ± 10.94 vs 33.01 ± 13.57; *t* = 1.206, *P* = 0.232) and CNFL (13.74 ± 2.19 vs 14.53 ± 3.19; *t* = 0.960, *P* = 0.340) were comparable between PD patients with and without pupillary oversensitivity. Furthermore, patients with pupillary oversensitivity were all from the AutD-S and AutD-M groups and had a higher SCOPA-AUT score (17.59 ± 6.68 vs 8.94 ± 7.72; *t* = 4.151, *P* < 0.001) than patients without oversensitivity. After adjustment for SCOPA-AUT there was no significant difference in CNFD (*P* = 0.878), CNBD (*P* = 0.801), or CNFL (*P* = 0.237) between the two groups.

### ROC analysis

**Fig. 5:**
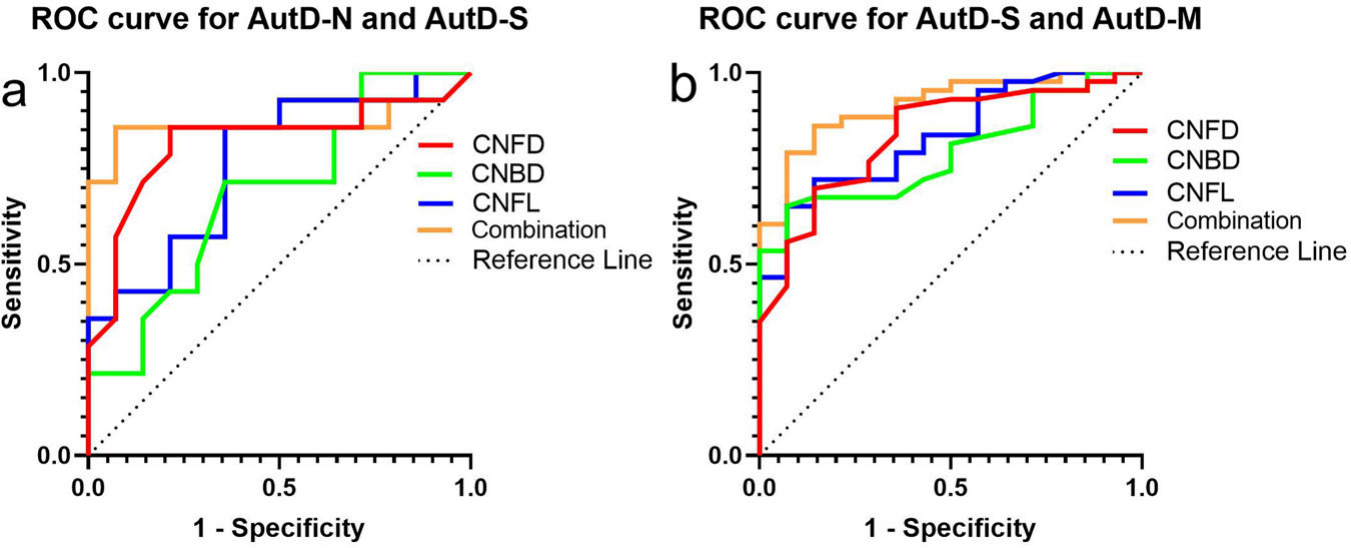
The ROC for CCM to distinguish AutD-N from AutD-S and AutD-S from AutD-M. **a**, **b** PD Parkinson’s disease, AutD-N patients with no autonomic symptoms, AutD-S patients with single-domain autonomic symptoms, AutD-M patients with multiple-domain autonomic symptoms, CNFD corneal nerve fiber density, CNBD corneal nerve branch density, CNFL corneal nerve fiber length, AUC area under the curve.

ROC analysis showed that CNFD, CNBD, and CNFL could distinguish between AutD-S and AutD-N with an area under the curve (AUC) of 81.89% (95% CI, 64.75–99.02%), 68.37% (95% CI, 48.45–88.28%), and 76.53% (95% CI, 58.72–94.34%), respectively. Using a CNFD cutoff of <29.84 no./mm^2^, the sensitivity and specificity for AutD-S was 85.71% and 78.57%. Using a CNBD cutoff of <37.94 no./mm^2^, the sensitivity and specificity for AutD-S was 71.48% and 64.29%. Using a CNFL cutoff of <17.06 mm/mm^2^, the sensitivity and specificity for AutD-S was 85.71% and 64.29%. A combination of all three corneal nerve parameters increased the AUC to 87.24% (95% CI, 58.72–94.34%), with a sensitivity and specificity of 85.71% and 92.86% (Fig. 5a).

The AUC distinguishing AutD-M from AutD-S for CNFD, CNBD, and CNFL was 83.72% (95% CI, 72.34–95.10%), 79.07% (95% CI, 67.42–90.72%), 83.31% (95% CI, 72.36–94.25%), respectively. Using a CNFD cutoff of <25.83 no./mm^2^, the sensitivity and specificity for AutD-M were 69.77% and 85.71%. Using a CNBD cutoff of <29.18 no./mm^2^, the sensitivity and specificity for AutD-M were 65.12% and 92.86%. Using a CNFL cutoff of <13.91 mm/mm^2^, the sensitivity and specificity for AutD-M were 65.12% and 92.86%. A combination of all three corneal nerve parameters increased the AUC to 91.53% (95% CI, 83.99–99.06%) with a sensitivity and specificity of 79.07% and 92.86%, respectively (Fig. 5b).

## Discussion

In the present study, we show that corneal nerve loss is associated with the severity of autonomic symptoms in patients with PD. Autonomic symptoms are an important and under-recognized area of functional disability that can severely affect the quality of life in patients with Parkinson’s disease. Delayed gastric emptying can lead to impaired drug absorption with the “delayed ON” or even “no ON” phenomenon interfering with the therapeutic effect of dopaminergic medication, worsening motor function. Orthostatic hypotension can cause syncope and falls^16^ and fall-related fractures, pneumonia, and even death. Some autonomic symptoms, such as constipation, can occur in the early stage of disease and may even precede the onset of motor symptoms by many years^16,17^. We speculate that both dopaminergic and adrenergic neurons in the nigrostriatal and peripheral nervous systems are lost progressively with the gradual worsening of motor and non-motor symptoms. Moreover, autonomic nerve dysfunction has been associated with faster disease progression and shorter survival^18^, thus timely and accurate detection of autonomic deficits is important for PD prognosis and management.

Autonomic nerve fibers are thinly myelinated or unmyelinated nerve fibers, but damage to these fibers is difficult to quantify. Pathological examination of skin biopsies has shown decreased intraepidermal nerve fiber density (IENFD)^19^ and a relationship between mean axonal length and total nerve fiber length with motor and autonomic symptoms and autonomic dysfunction^20^. Additionally, there is evidence of a non-length dependent distribution of phosphorylated α-synuclein in autonomic fibers in the skin of patients with PD^21,22^ with differences between patients with PD and multiple system atrophy^23^. While skin biopsy provides important insights in the study of autonomic neuropathies, it is invasive and requires complex immunostaining protocols in specialized laboratories^24,25^. Quantitative sensory testing (QST) is non-invasive and easily performed, but is subjective and can be highly variable. Indeed, a recent deep phenotyping study using the standardized German Research Network on Neuropathic pain protocol showed no differences between controls and drug-naïve PD patients^26^.

Cardiac ^123^I-MIBG scintigraphy^27^ and intestinal ^11^C-donepezil PET/CT^28^ can identify sympathetic denervation and impairment of parasympathetic terminals in PD patients, but these techniques are expensive and not readily available.

In this large cohort of patients with PD we show evidence of a proximal loss of corneal nerves as evidenced by a progressive reduction in CNFD, which was associated with the severity of autonomic symptoms. This is consistent with the findings in our small pilot study in 26 PD patients, where we also showed a correlation between corneal nerve loss and autonomic symptoms (SCOPA-AUT) and function (Deep breathing-Heart rate variability)^13^. Indeed, in the present study, we show that PD patients with pupillary oversensitivity have a lower CNFD, which is no longer significant after adjustment for the SCOPA-AUT score, further supporting a relationship between CCM and autonomic abnormalities. Corneal nerve loss has been associated with autonomic neuropathy in amyloid neuropathy^29^, fibromyalgia^30^, and diabetic neuropathy^12,31^. The increase in corneal nerve branches and length has been found in several previous studies in patients with PD^13,15^ and may represent nerve regeneration, especially in the earlier phases of the disease, as evidenced by the higher CNBD and CNFL in the AutD-N and AutD-S groups compared to the AutD-M group. To further assess the interplay between proximal nerve degeneration and distal nerve regeneration, we quantified the CNBD/CNFD ratio. While there was a trend for a decrease in the ratio with increasing severity of autonomic symptoms, this was not significant, suggesting a dynamic and complex process that requires careful interpretation in future studies.

We found no correlation between SCOPA-AUT and LEDD, suggesting that autonomic involvement in PD reflects intrinsic neurodegeneration and is consistent with our recent study, where we also showed no association between CNFD and LEDD^15^.

Corneal nerve loss assessed using CCM is evident even in patients without autonomic symptoms indicating sub-clinical deficits detected using CCM, which then progress with increasing severity of autonomic neuropathy. Indeed, CCM detected corneal nerve loss when IENFD was still normal in drug-naïve patients with Parkinson’s disease^32^. Previous studies suggest that the SCOPA-AUT scores correlated with age^33,34^, disease duration^34^, disease severity^35^, and cognitive function^36^. Therefore, we adjusted these confounders in the relation analysis between autonomic function and CNFD. Our study shows SCOPA-AUT related positively to CNFD.

The relatively good diagnostic outcomes to differentiate patients with minimal and more prominent autonomic symptoms from those without autonomic symptoms using individual and especially combined corneal nerve parameters argues in favor of the diagnostic utility of CCM in patients with PD and autonomic deficits. This adds to the diagnostic utility of CCM in PD as we have also recently shown that it has a good ability to differentiate PD patients with and without cognitive dysfunction^15^. Although this is a good-sized cohort of patients with PD, the cross-sectional design cannot infer causality. Longitudinal studies are required to assess if CCM can identify patients with a faster deterioration of autonomic symptoms and poorer prognosis as has been shown for motor progression recently^14^. We acknowledge that SCOPA-AUT is a subjective symptom questionnaire^36–38^, although studies have shown that it reflects the severity of autonomic dysfunction in PD^39,40^. We also acknowledge that the presence of cognitive dysfunction may limit the accuracy of the assessment of SCOPA-AUT.

In conclusion, this study shows an association between corneal nerve loss assessed using CCM with the presence of autonomic symptoms and an excellent diagnostic utility for identifying PD patients with autonomic symptoms. Therefore, CCM represents a safe, rapid, and convenient in vivo ophthalmic imaging technique to identify patients with PD and autonomic symptoms. These findings warrant longitudinal studies to define the prognostic utility of CCM in PD.

## Materials and methods

### Subjects

The study was approved by the ethics committee of Henan Provincial People’s Hospital. Patients with PD were recruited from Henan Provincial People’s Hospital between March 2017 and January 2020. All subjects agreed to participate in the study, and written informed consent was obtained.

Age and sex were assessed in all subjects. PD was diagnosed according to the 2015 Movement Disorder Society clinical diagnostic criteria for Parkinson’s disease^41^. Clinically established PD and clinically probable PD were included. Atypical parkinsonism such as progressive supranuclear palsy, cortical basal ganglia degeneration, multiple system atrophy, and secondary parkinsonism (drug-induced, immune-mediated, inflammatory, vascular, infectious, traumatic or neoplasm, etc.) was excluded from the study. Healthy controls were included from either volunteers or spouses of PD patients who had no history of movement disorder or cognitive impairment. For the investigational purpose, patients or healthy controls younger than 40 or older than 85 years of age were excluded from the study. Participants with a history of eye surgery, eye inflammation, glaucoma, corneal disease, or thyroid eye disease were excluded. Other causes of peripheral neuropathy were excluded by a history of excess alcohol use (>150 ml/day) and an assessment of vitamin B_12_ and folate, serum electrophoresis to exclude multiple myeloma, cryoglobulinemia, macroglobulinemia, and oral glucose tolerance test to exclude impaired glucose tolerance and diabetes. To increase diagnostic accuracy, the clinical profiles of each participant were carefully reviewed by two experienced neurologists (J.-J. Ma and H.-Q. Yang) who specialized in movement disorders.

### Clinical evaluation

The evaluation of motor and non-motor symptoms was all performed in the “ON” state in PD patients. Motor function was assessed with part I, II, III, and IV sub-scales of the unified Parkinson’s disease rating scale (UPDRS), and Hoehn and Yahr (H-Y) staging was undertaken for all patients^42^. Disease duration was defined as the time between presentation with first motor symptoms and enrollment into the present study. Montreal cognitive assessment (MoCA, Beijing Version) was used to assess the cognitive status. Anxiety and depressive symptoms were evaluated with the 14-item Hamilton anxiety (HAMA-14) rating scale and the 24-item Hamilton depression (HAMD-24) rating scale, respectively. Levodopa equivalent daily dose (LEDD) was assessed according to the levodopa conversion formula^43^. Briefly, 100 mg levodopa = 133 mg entacapone = 1 mg pramipexole = 5 mg ropinirole = 10 mg selegiline = 1 mg rasagiline = 100 mg amantadine.

### Autonomic symptom severity

The SCOPA-AUT, a reliable and validated questionnaire that evaluates autonomic symptoms, was undertaken in patients with PD^44^. Briefly, 27-items with six domain rating scales were used to evaluate autonomic symptoms (item 1–7 for the gastrointestinal tract, item 8–13 for urinary tract, item 14–16 for the cardiovascular system, item 17,18,20,21 for thermoregulation, item 19 for pupil activity, item 22–24 for male sexual function, item 25–26 for female sexual dysfunction; and item 27, treatment of either of above-mentioned symptoms). Each item is given a score, with a higher score indicating more severe autonomic dysfunction. PD patients were divided into three subgroups according to domain autonomic symptom^37^. PD patients with no autonomic symptoms were defined as AutD-N; PD patients with autonomic symptoms in one domain were defined as AutD-S; PD patients with autonomic symptoms in two or/more domains were defined as AutD-M. Orthostatic hypotension was defined as a drop of systolic blood pressure (≥20 mm Hg) or diastolic blood pressure (≥10 mm Hg) within 3 minutes of standing from a supine position^45^.

### Corneal confocal microscopy

A Heidelberg Retina Tomograph III with a Rostock Cornea Module (HRT III RCM; Heidelberg Engineering GmbH, Heidelberg, Germany) was used to acquire images of the central corneal sub-basal nerve plexus. Topical lidocaine was used to anesthetize the eye of each subject, and they were seated comfortably and instructed to fixate on an outer fixation light. The TomoCap was correctly positioned on the apex of the cornea by visualizing it with the CCD camera. An experienced examiner took images at the level of the sub-basal nerve plexus in the central cornea using the “section” mode according to an established protocol^46^. Four to six best-quality CCM images from the central cornea of each eye were selected and analyzed using a validated, manual (CCMetrics) and automated (ACCMetrics, Imaging Science and Biomedical Engineering, Manchester, UK) purpose-written software^47^. Three parameters were analyzed: (a) corneal nerve fiber density (CNFD): the number of main nerve fibers per square millimeter; (b) corneal nerve fiber branch density (CNBD): the number of primary branches originating from the main nerve; and (c) corneal nerve fiber length (CNFL): the sum of the length of all nerve fibers per square millimeter. The CNBD/CNFD ratio was calculated to assess nerve regenerative capacity^15^.

### Data analysis

The normality of data was assessed by the Shapiro-Wilk test. For normally distributed variables, numbers are expressed as mean ± standard deviation (SD). Analysis of variance with Bonferroni as post hoc test was used for multiple group comparison. For non-normal or non-homoscedasticity variables, numbers are expressed as median (interquartile range). The nonparametric Kruskal–Wallis test was used for multiple comparisons. Chi-square tests and Fisher’s exact tests were used to compare categorical variables. Partial correlation analysis was performed in PD to assess the association between SCOPA-AUT scores and corneal nerve parameters and clinical characteristics, adjusting for confounders. The ROC curve was used to analyze the capability of CNFD, CNBD, and CNFL for distinguishing PD patients with single-domain autonomic impairment from no autonomic impairment, and multiple-domain autonomic impairment from single-domain autonomic impairment. All analyses were carried out using SPSS version 22.0 (IBM Corporation, Armonk, NY, USA). Dot plots and ROC curves were generated using GraphPad Prism version 8.0 (GraphPad Software, Inc, San Diego, CA, USA). *P* < 0.05 was considered statistically significant.

## Data Availability

The authors confirm that the data supporting the findings of this study are available within the article.
